# Disability and Psychiatric Symptoms in Men Referred for Treatment with Work-Related Problems to Primary Mental Health Care

**DOI:** 10.3390/healthcare5020018

**Published:** 2017-03-24

**Authors:** S. Kathleen Bailey, Christopher J. Mushquash, John M. Haggarty

**Affiliations:** 1 Department of Psychology, Faculty of Health and Behavioural Sciences, Lakehead University, 955 Oliver Road, Thunder Bay, ON P7B 5E1, Canada; cjmushqu@lakeheadu.ca (C.J.M.); haggartyj@tbh.net (J.M.H.); 2 St. Joseph’s Care Group, 710 Victoria Avenue East, Thunder Bay, ON P7C 5P7, Canada; 3 Northern Ontario School of Medicine, 955 Oliver Road, Thunder Bay, ON P7B 5E1, Canada

**Keywords:** collaborative care, men, outpatient, PHQ, WHODAS 2, work, mental health, primary care

## Abstract

The relationship between male sex and employment as barriers to accessing mental health care is unclear. The aim of this research was to examine (1) whether the clinical features of men referred to a shared mental health care (SMHC) service through primary care differed when symptoms were affecting them in the work domain; and (2) empirically re-evaluate the effectiveness of a SMHC model for work-related disability using a pre-post chart review of N = 3960 referrals to SMHC. ANOVA and logistic regression were performed to examine symptoms (Patient Health Questionnaire, PHQ) and disability (World Health Organization Disability Assessment Schedule, WHODAS 2) at entry and discharge. Men were RR (relative risk) = 1.8 (95% C.I.: 1.60–2.05) times more likely to be referred to SMHC with work problems than women. Having greater disability and more severe somatic symptoms increased the likelihood of a work-related referral. There were no significant differences after treatment. Problems in the work domain may play an important role in men’s treatment seeking and clinicians’ recognition of a mental health care need. This study is relevant because men are underrepresented in mental health (MH) treatment and primary care is the main gateway to accessing MH care. Asking men about functioning in the work domain may increase access to helpful psychiatric services.

## 1. Introduction

Gender disparities exist in patterns of mental illness, diagnosis, and treatment. Sex-based differences in help-seeking behaviour and in symptom recognition by health professionals account for many of these variations [1]. Men are much less likely than women to seek any type of help for psychiatric or other medical symptoms [2] and less likely to seek formal and professional psychiatric services, psychotherapy, and/or counselling in particular [3]. Although most Canadians access mental health services by means of their primary care (PC) provider [4], men make fewer visits to PC physicians and other medical services than women [5], thereby limiting men’s access to support for mental health-related symptoms and disability.

Employment may be an additional reason men seek less help for mental health issues. A population-based study found that men who were employed and experiencing mental health problems were significantly less likely than those who were unemployed to visit their PC physician about their symptoms, while women’s help-seeking was unrelated to employment status. Unemployed men and those on a sickness/disability absence were nearly three times more likely to seek help from their PC physician [6]. Unfortunately, the vast majority of published research on help-seeking has not examined the relationship between gender and employment status, and studies examining mental health and workplace disability have focused on sick-listed employees rather than at the time when they first access treatment and may still be working.

On the job performance, rates of illness, absenteeism, accidents, and staff turnover are all affected by employees’ mental health status. Overall, one in five Canadian adults is affected by high levels of self-reported psychological distress (men: 19.23%, women: 22.20%) [7] and more than one in six (17%) reported a need for mental health care. Symptoms consistent with a major depressive episode, a substance use disorder, generalized anxiety disorder, or bipolar disorder affected as many as 1 in 10 Canadians over the age of 15 [8]. In the general population, men’s 12-month and lifetime prevalence of any mental health or substance use disorder was higher than women’s (12-month: 10.6% and 9.6%; lifetime: 38.1% and 28.2%, respectively) [9].

Common mental disorders may be even more prevalent in the working population. Over 8.5% of a large sample of Canadian workers had experienced a depressive episode in the previous year [10], compared with 4.7% in the general Canadian population [8]. Given such pervasiveness of common mental disorders in workers, and the individual and societal consequences of mental disorders, it is perhaps surprising that only approximately 9.5% of all Canadians access specialized mental health services each year [4], and only half of workers who had a moderate or severe depressive episode [10]. Most Canadians with a common mental disorder pursued help through PC [11], making it an important setting for research into mental health care access.

Stigma around mental illness and treatment plays an important role in men’s treatment-seeking behaviour. Men with a mental illness were more likely than women to report feeling embarrassed about their problems and to experience discrimination because of their symptoms [11]. Adding to that, men who reported feeling embarrassed about their symptoms were nearly seven times less likely to seek help from their PC provider compared with men who did not report significant embarrassment [6]. Not only do men report higher levels of perceived stigma about mental illness, they also display higher levels of personal stigma toward those treated for mental health problems compared to women [12] and greater amounts of self-stigma (lowered self-esteem), than women over their mental health symptoms [13]. The negative effects of mental health-related stigma can be long-lasting, affecting men well into and beyond recovery even when they do receive treatment. Men who entered treatment with comorbid mental disorder and substance abuse were just as likely after one year of treatment, and a significant decrease in their symptoms, to expect stigmatization based on their mental health problems. About two thirds of men in the study expected interpersonal rejection, to be looked down upon, and disadvantage in employment situations because of their mental health difficulties at baseline. After one year of treatment and significant clinical gains, the expectations of these men in terms of stigma had not changed significantly [14]. Too often though, men are deterred from any mental health treatment-seeking because of stigma [13]. This may be particularly relevant for men with less severe symptoms of common mental disorders, as help-seeking and treatment participation might be seen as optional, and therefore more of a threat to self-esteem. Providing mental health care in a less stigmatizing environment, such as through a PC provider, may improve treatment-seeking and utilization for men who are experiencing mental health symptoms and related disability on the job.

The consequences of untreated mental health problems are wide-ranging. Psychological impairment and distress decrease worker productivity [15]. Psychiatric symptoms increase men’s risk of economic loss through unemployment and lack of advancement, which can expose a person to even higher levels of psychological distress and disorder. As Addis and Mahalik [16] pointed out, improving men’s health behaviours, such as treatment seeking when experiencing symptoms of impaired wellbeing, is one obvious avenue improving men’s lives overall.

The economic impact of mental disorders is mainly a result of unemployment, decreased productivity, and disability [17]. The principal cause of long-term absences from work and of disability allowances paid in Canada was mental health problems (estimated between 30% and 50%) [18]. Even minor psychiatric disorders and impairments have been linked to increased absences from work, and the presence of psychiatric symptoms reduces the likelihood of a timely return to work after taking a leave [19], particularly when symptoms are more severe [20]. Longer work absences are associated with reduced likelihood of ever returning [21], whereas earlier interventions are significantly associated with a shortened disability episode [22]. Therefore, identifying workers with psychological symptoms early on, and recognizing appropriate service models that can effectively relieve their symptoms is likely to benefit workers and employers by respectively lessening their aggregate emotional and economic burden.

Shared mental health care (SMHC), where psychiatrists and other mental health care providers are co-located and work collaboratively with PC physicians to provide intervention and support, is a promising model. It was hypothesized that SMHC enhances accessibility of mental health services in part by reducing stigma [23] and by improving timely access to care [24]. Counseling needs are the least likely mental health needs to be met, and men were less likely than their female counterparts to describe having a mental health care need that was met (males: 8.8%; females: 14.4%) or partially met (males: 2.7%; females 4.6%) [25].

The purpose of this study was to clarify the clinical features of men referred for mental health services through PC, with an emphasis on those whose symptoms were affecting them in the work domain. Specifically, this study aimed to fill a gap in the literature by (a) identifying the types of problems and symptoms perceived by care providers when a referral to SMHC was made for work- or employment-related reasons; (b) examining the symptoms and impairment men with work domain referrals reported experiencing when they presented in SMHC, and (c) empirically re-evaluating the effectiveness of a SMHC model for men with work- and employment-related disability. Bettering clinicians’ understanding and recognition of symptoms that lead to men’s mental health treatment when symptoms impede work, and the characteristics of a successful approach to treatment, can improve access to appropriate and efficacious care.

## 2. Materials and Methods

This study was a sex-based gender analysis, meaning that male sex was a selection criterion for inclusion in this subanalysis; however, interpretation was based on consideration of male gender as a social and psychological construct affecting men’s experiences and behaviour. This project was reviewed and approved by the St. Joseph’s Care Group Research Ethics Board.

All data collection took place between July 2001 and June 2011 (N = 3960 men and women, aged 17–75 years). The SMHC service where data was collected is located within an urban (population >100,000) but geographically isolated (closest similarly-sized community is about 700 km away by road) PC clinic in northern Ontario, Canada and serves approximately 18,000 patients. At a local level, Statistics Canada provided that the population in 2011 was 50.5% female, and that the provincial employment rate for those over 15 years of age is on par with the national average, at 65% and 57% for men and women, respectively. Referrals for psychiatric consultation or counseling were predominantly from any of the approximately fifteen (there was some variability over the study period, up to 19) PC physicians practicing out of the clinic, although other on-site allied health professionals could also make referrals (nurse practitioner, registered nurse, social worker, dietician). Throughout the study period, the clinic employed two full time equivalent counsellors (Social Workers) and the Psychiatrist was in-clinic one half day per month and available for consultation on an ongoing basis. Services provided at the clinic are entirely paid for by the provincial health insurance program. PC providers can be accessed on an as-needed basis and the wait time for counselling services was about 42 days [24]. Private (i.e., not publicly funded) mental health care services are available for those who have private insurance or are willing to pay out of pocket. Most publicly funded mental health programs are available exclusively by referral from a primary health care provider. The demographic variables and other measures used for this study are part of the standardized referral form and intake procedure given to all clients accessing the mental health service, as such this study constitutes a secondary data analysis. Patients who attended three or more treatment sessions were invited to repeat the measures before discharge. The rationale for collecting exit measures only from patients who engaged with the service was to increase the probability that measured change was a result of intervention. This decision was made at the time the clinic opened, thus data from those who attended fewer than three appointments was not available for the retrospective, longitudinal analysis. Since its inception, this service upheld a philosophy of quick access to care and continuous quality improvement through empirical research. As part of that commitment, the nature of the ongoing research was explained to all clients verbally and in the form of a letter during their initial appointment at the mental health clinic, at which point verbal consent for participation was given. Patients were given the option not to participate in research or withdraw at any time without consequence. The dataset used for research purposes was anonymized, contained no patient names or other identifiers which could be used to identify individuals, and was housed on a separate network within the health centre. Non-participation with initial survey completion was less than 5% at the initial appointment and did not affect access to care. Nevertheless, as is often the case in applied health research, incomplete and missing data was not uncommon.

From the overall SMHC sample (both men and women), those who were identified on the mental health services referral form as currently experiencing work-related problems (*n* = 633), problems to do with unemployment (*n* = 215), or a workplace disability claim (*n* = 27) were separated to form the “work group,” N = 784, or 19.8% of SMHC sample. Unfortunately, the space on the referral form where clinicians could record the patient’s employment type and status was seldom used, and so no further employment details were available to the researchers.

### 2.1. Instruments

Demographic information and reason(s) for referral were provided on the SMHC referral form. The referring clinician provided patients’ demographic and contact information as well as information related to the reason for referral in a check box format. Clinicians were asked to identify as many relevant Psychiatric Symptoms (e.g., depressed mood, obsessive thoughts, phobia, alcohol abuse) and Psychosocial Issues (e.g., separation/divorce, self-esteem, bereavement, work problems) for the current episode as they felt necessary to capture the difficulties of the client.

World Health Organization Disability Assessment Schedule (WHODAS 2). The WHODAS 2 is designed to measure and quantify the difficulties a person experiences due to health conditions (disease, illness, injury, mental health, emotional problems, and problems with drugs or alcohol). The simple sum scoring method was proposed and tested by Andrews and colleagues [26] and found to have good reliability and validity in clinical and community samples of adults. Sum scores for global disability range from 0 = no disability to 48 = complete disability. The WHODAS 2 was adopted in the Diagnostic and Statistical Manual of Mental Disorders (5th ed.; DSM-5) and is recommended as part of the diagnostic interview. The internal consistency of the WHODAS 2 in our sample was good, α = 0.88.

The Patient Health Questionnaire (PHQ), PHQ-9, and PHQ-15. The PHQ is a self-administered screening tool for making provisional diagnoses and monitoring of selected DSM-IV disorders common to PC settings, including depressive, anxiety, somatic symptoms, and alcohol use. The PHQ has demonstrated excellent validity and reliability, and a high level of diagnostic agreement with mental health professionals [27]. The PHQ-9 utilizes nine items from the full PHQ for monitoring and measuring the severity of depression symptoms. Scores range from 0 to 27. Scores of 5, 10, 15, and 20 represent mild, moderate, moderately severe, and severe depression, respectively. PHQ-9 has high specificity and sensitivity for major depression and was validated in the PC setting [28]. Reliability analysis for PHQ-9 in our sample revealed a high level of internal consistency, α = 0.99. Similarly, the PHQ-15 measures somatic symptom severity and demonstrates good psychometric properties in PC [29]. Scores range from 0 to 30. Scores of 5, 10, and 15 represent low, medium, and high somatic symptom severity, respectively. Again, internal consistency in our sample was very high, α = 0.99. PHQ-9 and -15 are endorsed in the DSM-5 to enhance clinical decision-making.

### 2.2. Statistical Methods

All analyses were performed using SPSS 21.0 (IBM, Armonk, NY, USA), with the exception of the missing data analysis, which was completed using SPSS 22.0. All reported *p*-values are two-tailed.

Descriptive statistics and *t*-tests were used to compare the sample characteristics and demographic variables of men in the work group and non-work group. Clinical features and treatment response of men in the two groups were compared using one-way ANOVA on baseline and exit measures. Logistic regression was carried out to ascertain whether global disability, depression severity, and somatic symptom severity could predict referrals to SMHC with problems perceived to be work- and non-work related. Specifically, we performed logistic regression to test a model for predicting referral group membership (work group or non-work group). Baseline total disability (WHODAS 2) was entered as a continuous variable and baseline depression severity (PHQ-9) and somatic symptom severity (PHQ-15) as categorical measures with no symptoms as the referral category. To be thorough, we repeated the logistic regression by entering the raw scores from the two PHQ measures as continuous variables.

There were significantly fewer records at exit than baseline. Descriptive statistics were inspected and one-way ANOVA was performed using the presence or absence of any completed exit measures as the independent variable and with age, baseline PHQ-9, baseline PHQ-15, and baseline WHODAS 2 as the dependent variables.

### 2.3. Informed Consent

All procedures followed were in accordance with the ethical standards of the responsible committee on human experimentation (institutional and national) and with the Helsinki Declaration of 1975, as revised in 2013, Informed consent was obtained from all patients for being included in the study.

## 3. Results

Overall, results indicated clinically and statistically significant differences between men referred from primary care for mental health treatment with and without problems identified in the work domain.

### 3.1. Characteristics of Participants

Less than one third (29.4%, *n* = 1164) of the entire sample was male. There was a higher percentage of men in the work group 43.0% (*n* = 337) compared with 26.0% (*n* = 827) in the non-work group. Chi square analysis confirmed the independence of these samples, χ^2^ (1, N = 3960) = 87.00, *p* < 0.001. Having a work-related referral to SHMC was almost twice as likely for men as for women, demonstrated by the relative risk (RR) of 1.81 (95% C.I.: 1.60–2.05).

Mean age did not differ significantly between men in the work (*n* = 337; 39.61 years) and non-work (*n* = 827; 39.23 years) groups. A greater number of psychological and psychosocial reasons for referral were identified on the SMHC referral form of men in the work group. The mean number of psychiatric symptoms identified was 2.55 (range: 0–7) in the work group and 1.96 (range: 0–8) in the non-work group, t = 6.106, *p* < 0.001. The mean number of psychosocial reasons was 3.07 (range: 1–8) in the work group and 1.29 (range: 0–8) in the non-work group, t = 16.662, *p* < 0.001. Mean number of treatment visits to the SMHC clinic was about three and did not differ in a statistically significant way between groups (M = 2.87, SD = 3.41 in the work group and M = 3.06, SD = 3.85 in the non-work group, n.s.). Treatment visits included those visits made following the initial referral visit in PC and an intake appointment where initial interview, consent procedures, and baseline measures were completed. In the work group, 61.4% (range: 0–22) of men attended between one and five treatment visits, compared with 60.5% in the non-work group (range: 0–47).

### 3.2. Clinical Features at Baseline and Exit

The most common disorders according to the PHQ were all more prominent in the work group than the non-work group at baseline, with statistically more patients in the work group reporting clinically heightened scores on somatic disorder and major depressive disorder, and non-significant differences in “panic”, “other anxiety”, and “alcohol abuse”. On exit measures, only “other anxiety” was significantly more likely in the work group (*p* = 0.04), although symptoms were not clinically significant on that scale or any of the others in either the work group or the non-work group.

One-way analysis of variance (ANOVA) showed that men’s total disability scores and depression severity and somatic symptom severity were all significantly higher at baseline in the work group than in the non-work group. Homogeneity of variance between the two groups was confirmed for WHODAS 2, PHQ-9, and PHQ-15 with Levene’s statistic. Further details, including effect size, can be seen in Table 1.

The simple summation method of scoring does not take into account the final three questions of the WHODAS 2, leaving out important information that appears to be specifically relevant to occupational functioning. These items asked participants to indicate the number of days in the previous 30 days that (a) your difficulties were present; (b) you were totally unable to carry out your usual activities or work because of any health condition; and (c) not counting the days that you were totally unable, for how many days did you cut back or reduce your usual activities or work because of any health condition. We examined men’s responses to these items individually to see whether men referred for problems related to work differed significantly from those who were referred to SMHC for other reasons (Table 2).

**Figure 1:**
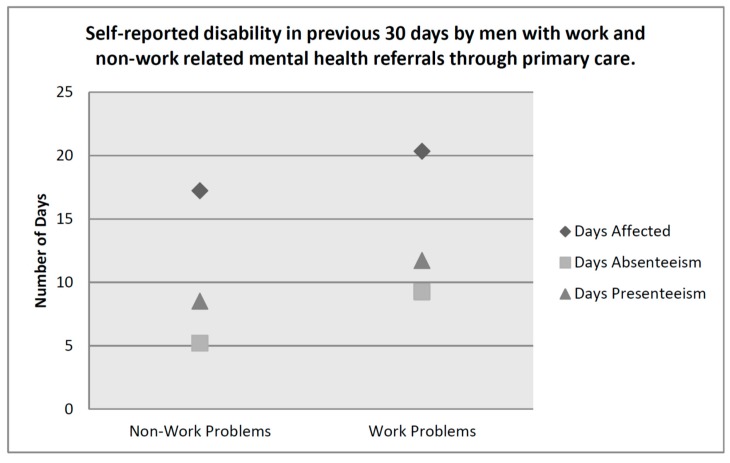
Self-reported disability in previous 30 days by men with work and non-work related mental health referrals through primary care (PC).

Figure 1 demonstrates that men who sought treatment for work related mental health (MH) complaints in PC had more self-reported disability than their non-work group counterparts at the time of referral. A comparison of those who completed the exit questionnaires from the work and non-work groups revealed no significant differences in total disability (WHODAS 2), depression severity (PHQ-9), or somatic symptom severity (PHQ-15) at discharge from SMHC (Table 3).

### 3.3. Predicting Work-Group Membership

Several cases were excluded from the logistic regression analysis due to missing data, leaving *n* = 381 (32.7%) of N = 1164 cases in the analysis. Results of the Lagrange multiplier test indicated that all three variables were expected to improve the model’s fit, which was indeed the case, χ^2^ = 26.445, df = 8, *p* < 0.001. This means that the predictors, taken together, distinguished reliably between groups. The non-significant Hosmer and Lemeshow test demonstrated that model prediction did not differ significantly from the observed pattern. Prediction success overall was not improved by the addition of the WHODAS 2 and PHQ-9 and -15, and remained at 72.2% accuracy with and without the addition of disability scores and symptoms severity, however, classification in the work group improved from 0% to 7.5% correct. Only disability made a significant contribution to predicting referral group (Wald = 5.240, *p* = 0.022), with the odds of being in the work group increasing by 4.3% with every one point increase in WHODAS 2 scores (Exp(B) = 1.043, 95% C.I. = 1.006–1.082).

After repeating the analyses using raw scores, again, all three variables improved the model’s fit as predicted in the first step, χ^2^ = 25.551, df = 3, *p* < 0.001 and the Hosmer and Lemeshow test was not significant, indicating a good fit of the model. In this model both somatic symptoms and disability were significant to the prediction of work-related MH referrals. A one-point increase in PHQ-15 led to a 6.4% increase in the odds of having a work-related MH referral, (Exp(B) = 1.064, 95% C.I. = 1.002–1.129, *p* = 0.042) and a one point increase in WHODAS 2 increased the odds by 4.2%, (Exp(B) = 1.042, 95% C.I. = 1.005–1.081).

### 3.4. Missing Data Analysis

Missing data analysis revealed that there were no significant differences in the baseline clinical indicators (WHODAS, PHQ-9, PHQ-15) between men who completed the follow up and those who did not, either in the entire dataset nor in the work group only subset. Mean age was slightly higher in the entire sample and subsample of patients that provided follow up compared with those who did not provide follow up measures (M = 42 vs. 38 years).

## 4. Discussion

This study overcomes an important gap in the literature on mental health help-seeking among males. Although being male and being employed are known to be related to decreased help-seeking, published studies to date have rarely included adequate detail to delineate the effect of sex on the employment and help-seeking relationship or did not measure both variables. This study makes a contribution to our understanding of men’s mental health treatment seeking because we demonstrated a striking increase in mental health referrals when men’s symptoms were affecting them in a particular domain. Furthermore, our study provided empirically-based insight into which mental health symptoms contribute to men’s work-related disability and treatment-seeking through PC. One notable exception in the literature published to date was the study by Drapeau, Boyer, and Lesage [30] which demonstrated that male workers were less likely to seek treatment for their mental health problems than unemployed men and women with or without employment. The nature of that study precluded the researchers from examining the role of clinical symptoms or functional impairment when people did seek treatment. Men in our study were referred by PC providers who identified their mental health need as work- or employment-related. This is a useful contribution to the literature in that it provides an examination of when the men seen in PC are more likely to be recognised to possibly benefit from a mental health intervention.

Men in our study were almost twice as likely as women to be referred for mental health support by their PC provider because of problems related to work and employment. This represents a significant proportion of men who are referred to treatment for mental health symptoms. A possible interpretation of our findings is that clinicians recognised men’s complaints as potentially aided by mental health care more when they relate to functioning in the work domain. Establishing a better understanding the clinical characteristics and precursors that lead to referral may help provide more men with access to mental health care and thereby decrease suffering and consequences. Treatment outcomes are greater for people who are employed and the effect of employment on treatment response for mental health symptoms is greater for men than for women [31]. Identifying avenues to care for men who have mental health problems that interfere with employment should therefore be highly pertinent to patients, clinicians, employers, and insurers. SMHC appears to be one such avenue.

Men in the work group were more likely to meet criteria in all diagnostic categories that we examined on the PHQ than men in the non-work group, and the difference was significant for major depression and somatic complaints. Additionally, clinicians identified more psychiatric symptoms and psychosocial reasons for the referral when referrals also included a work-related reason. Comorbid and single common mental disorders (e.g., depression, anxiety, substance use disorders) pose a greater risk for long term absence from work than physical illness [32]. Our results imply that many men are still at work, but not functioning optimally, despite having multiple mental health symptoms.

Functional impairment, as measured by the WHODAS 2, was significantly higher in the work group at time of referral. The work group mean score of 14 on the WHODAS 2 at baseline was above the 85th percentile for individuals with any mental disorder, 90th percentile for individuals with one mental disorder, and above the 75th percentile for individuals with more than one mental disorder, according to published norms based on N = 8841 Australian participants. Following treatment, men in both groups (work and non-work) still had WHODAS 2 scores above the population mean for men, but similar to those for people with a mental health condition in the previous year [26]. Not only did men with work-related problems experience greater global disability, they reported feeling the effects of their symptoms a greater number of days in the previous month, and had more days when they were either totally or partially unable to carry out their normal activities because of their symptoms. To the extent that being totally unable to carry out normal activities can be equated with absenteeism, both clinical groups reported very high rates of this type of disability. Absenteeism rates in a nationally representative sample of healthy American workers were far below those reported in our study, at 5.1 days per year [33]. These results are meaningful for those interested in worker absenteeism and presenteeism due to mental health-related disability. It may suggest that when mental health symptoms interfere with work-related functioning, some men may become more likely to report their problems to a PC provider.

Somatic symptoms appear to play an important role in men’s referrals to SMHC. Although the proportion of variance in referral reasons explained by the PHQ-15 was small, somatic symptom severity as a continuous variable did play a significant role in predicting reason for referral using logistic regression. Limitations in physical activities due to mental health symptoms was previously identified as an important predictor of men’s help-seeking from their PC provider [6], and the relationship appears to be stronger for men referred with work-related impairment. Somatic symptoms play a prominent role in the experience of depression and anxiety [34]; however, men in PC were at least 50% less likely than women to report common physical symptoms, regardless of the presence or absence of psychiatric comorbidity [35]. It might be that men find physical symptoms as more acceptable reasons for seeking help versus typical mental health symptoms, or that men’s experience of mental health symptoms are atypical to begin with [36]. That may explain why somatic complaints alone, and not clinically significant depression or anxiety symptoms, contributed to predicting mental health referrals. Men with depression were found to be less articulate about their symptoms [37] and to focus more on external factors causing them distress [38]. Finally, it is possible that the men in this study experienced fewer psychological symptoms when compared to females, and that their mental health distress was expressed through physical symptomology. A longitudinal study examining characteristics of treatment-seekers and –seeking could elucidate whether there is a tipping point for men with mental health problems; when somatic symptoms and disability impact workplace functioning, perhaps some men are more willing to seek professional support. Further, it might be that the less stigmatizing environment offered by SMHC contributes further to increasing help-seeking behaviour when people are otherwise reluctant to do so.

Important limitations in the data collection phase of this study need to be acknowledged. First, a single clinic contributed data over a ten-year period. It is possible that the SMHC clinic where data collection took place was demographically unique. During the first half of the study period, this was the only SMHC clinic in the community. In previous studies, we examined referrals from up to five traditional mental health services in this same community and the second large SMHC clinic that later opened, and found no differences in sex or age of patients seen in SMHC [24,39], suggesting these patients were not demographically unique. Second, exit measures were only collected from patients who attended three or more treatment sessions. The decision to collect clinical outcome measures on such a schedule was made before the clinic opened and was admittedly less than ideal, in hindsight, from a research perspective. As is often the case in applied health research, critical research-relevant decisions are made in the absence of experienced researchers. As clinical researchers, we believe these data to be robust enough to stand up to these weaknesses, when limitations are thoroughly and clearly communicated and considered when interpreting results.

A further limitation in this study was that we did not have access to more details related to patients’ employment status and employment-related outcomes and changes. Although more than two thirds of the work sample were specifically referred for problems at work, we cannot definitely conclude that these men were employed at the time of referral and end of treatment any more than we can conclude that those referred with problems related to unemployment had remained unemployed, or that those referred with workplace insurance claims were absent from or returned to work. We recognise that a clinically significant reduction in mental health symptoms is not always sufficient to translate to a recovery of work-related functioning, and recovery from mental health-related functional disability may take longer than recovery from affective impairment. A clear strength of our study was that we measured and demonstrated reductions in psychiatric symptoms and self-reported levels of disability following treatment.

## 5. Conclusions

This was the first study, to our knowledge, that attempted to assess the outcomes of SMHC for patients who were not exclusively already on disability absence. In fact, the only published study examining the effect of a SMHC model for sick-listed workers [40] cited low treatment fidelity in their return-to-work intervention and did not adhere to the optimal SMHC model by having case managers with a mental health background [41], as was the case in our study. The assessment and intervention provided in the SMHC clinic was as effective for men in the work and non-work groups, according to outcome measures. These results demonstrated that men in our sample with work-related mental health problems were referred with a greater number of symptoms, were more likely to have clinically significant impairment and higher levels of disability, but were as likely as men with non-work referrals to indicate they experienced symptom relief and lowered disability following a small number of treatment sessions. Unlike Vlasveld and colleagues [40], we did not examine return to work as an outcome, however, we do feel that our results demonstrate the SMHC model can be an effective intervention for men with mental health problems that are impacting employment and employability.

We utilized a sex-based gender analysis approach for this study, that is, one that selected cases based on the binary sex categorization commonly used in medical settings but conceptualized behaviour and sociological influences from a gender constructivist perspective. Although sex and gender are highly correlated, we attempted thoughtful consideration of the social and psychological contributions to men’s experiences and behaviour. We found this to be a useful methodology for conducting research and interpreting these specific findings in a clinically relevant way. This was a longitudinal observational study, and as such, we cannot be sure of any direction of causality. Integrating a gender constructivist perspective into our analysis of the findings provided a framework, albeit one with inherent biases around the social construction of gender and interplay between societal roles and expectations among men and women and access to the mental health care system. It is our opinion that we have just scratched the surface of possible contributing factors to patterns of mental health care access seen in men, and we hope to see a further expansion of this model by other researchers in the future.

## Figures and Tables

**Table 1:** One-Way ANOVA of baseline disability, depression, and somatic symptom severity in men referred to shared mental health care (SMHC) via primary care (PC).

Instrument	Work Group Mean (SD) 95% C.I.	Non-Work Group Mean (SD) 95% C.I.	F (df)	M.S. (Error)	Eta Sq (η^2^)
WHODAS 2	14.42 (8.36) 13.23–15.61	10.80 (8.18) 10.08–11.51	26.792 * (1, 690)	1814.55 (67.73)	0.038
PHQ-9	13.31 (6.92) 12.40–14.21	10.96 (6.71) 10.40–11.53	19.132 * (1, 766)	878.19 (45.90)	0.024
PHQ-15	10.76 (5.55) 9.80–11.71	8.50 (4.88) 7.97–9.03	18.533 * (1, 460)	478.20 (25.80)	0.039

Work group: World Health Organization Disability Assessment Schedule (WHODAS) *n* = 191; PHQ-9 *n* = 227; PHQ-15 *n* = 131. Non-work group: WHODAS *n* = 501; PHQ-9 *n* = 541; PHQ-15 *n* = 331. * *p* < 0.001. PHQ, Patient Health Questionnaire.

**Table 2:** Self-reported disability in previous 30 days by men with work and non-work related mental health referrals through PC.

Self-Reported Disability	Mean (Range)	S.D. (S.E.)	95% C.I.	F (df)	*p*-Value
Days affected					
Work Group	20.33 (0–30)	9.61 (0.693)	18.96–21.70	11.887 (1, 667)	0.001
Non-Work Group	17.22 (0–30)	10.90 (0.499)	16.24–18.20		
Days totally unable					
Work Group	9.24 (0–30)	10.97 (0.782)	7.7–10.78	25.641 (1, 691)	<0.001
Non-Work Group	5.19 (0–30)	8.85 (0.397)	4.41–5.97		
Days partially unable					
Work Group	11.72 (0–30)	10.96 (0.793)	10.15–13.28	13.526 (1, 667)	<0.001
Non-Work Group	8.51 (0–30)	9.88 (0.452)	7.62–9.39		

Work group: *n* = 191; Non-work group: *n* = 501.

**Table 3:** One-way ANOVA of disability, depression, and somatic symptom severity after treatment in men referred to SMHC via PC.

Instrument	Work Group Mean (SD) 95% C.I.	Non-Work Group Mean (SD) 95% C.I.	F (df)	M.S. (Error)	Eta Sq (η^2^)
WHODAS 2	7.05 (7.44) 5.78–8.33	6.04 (6.96) 5.45–6.64	2.175 (1, 660)	108.234 (49.774)	0.003
PHQ-9	4.88 (4.81) 4.07–5.70	4.44 (4.73) 4.05–4.83	0.972 (1, 697)	21.860 (22.494)	0.001
PHQ-15	5.81 (4.25) 5.01–6.61	5.46 (3.99) 5.09–5.82	0.680 (1, 577)	11.090 (16.304)	0.001

All ANOVAs were non-significant. Work group: WHODAS *n* = 133; PHQ-9 *n* = 137; PHQ-15 *n* = 110. Non-work group: WHODAS *n* = 529; PHQ-9 *n* = 562; PHQ-15 *n* = 469.

## Abbreviations

The following abbreviations are used in this manuscript:

SMHC	Shared Mental Health Care
PC	Primary Care
WHODDAS	World Health Organization Disability Assessment Schedule
PHQ	Patient Health Questionnaire
